# ASSOCIATION BETWEEN LATE LIFE EDUCATION AND COGNITIVE FUNCTION AMONG U.S. OLDER ADULTS

**DOI:** 10.1093/geroni/igad104.3190

**Published:** 2023-12-21

**Authors:** Nan Wang, Matthew Dupre, Hanzhang Xu

**Affiliations:** University of California, Davis, Sacramento, California, United States; Duke University, Durham, North Carolina, United States; Duke University, Durham, North Carolina, United States

## Abstract

This study assessed whether late-life education (LLE) was associated with better cognitive function and whether the benefits of LLE on cognitive decline differed by gender, race/ethnicity, and education level in a nationally-representative sample of U.S. older adults. We conducted a retrospective cohort study using six waves of data from the Health and Retirement Study ([HRS] 2008-2018) that included adults aged ≥ 65 with no baseline diagnosis of Alzheimer’s disease and related dementias (ADRD). Cognitive function was measured at baseline and over time using a summary score that included immediate/delayed word recall, serial 7’s test, objective naming test, backwards counting, recall of the current date, and naming the president/vice-president (range=0-35). LLE was measured at every wave and was categorized as “once a month or more,” and “not in the last month [or never].” Covariates included participants’ demographic background, socioeconomic status, psychosocial and behavioral factors, and health-related factors. Of 12,099 participants (median age 71 [IQR=10]), engaging in LLE at least once a month or more was associated with better cognitive function and was equivalent to a 2-4 year delay in cognitive decline than non-participation in LLE. The association was only partially attenuated after adjusting for a wide range of covariates. In addition, the benefits of LLE on cognitive function did not significantly differ by gender, race/ethnicity, or prior educational attainment. This study provides new insights into the protective role of LLE on cognitive function and underscores the importance of continued learning among older adults.

